# Development of Bone Targeting Drugs

**DOI:** 10.3390/ijms18071345

**Published:** 2017-06-23

**Authors:** Molly Stapleton, Kazuki Sawamoto, Carlos J. Alméciga-Díaz, William G. Mackenzie, Robert W. Mason, Tadao Orii, Shunji Tomatsu

**Affiliations:** 1 Department of Biological Sciences, University of Delaware, Newark, DE 19716, USA; mstaple@udel.edu (M.S.); robert.mason@nemours.org (R.W.M.); 2 Nemours/Alfred I. duPont Hospital for Children, Wilmington, DE 19803, USA; kazuki.sawamoto@nemours.org (K.S.); william.mackenzie@nemours.org (W.G.M.); 3 Institute for the Study of Inborn Errors of Metabolism, Pontificia Universidad Javeriana, Bogotá D.C. 110231, Colombia; cjalmeciga@javeriana.edu.co; 4 Department of Pediatrics, Graduate School of Medicine, Gifu University, Gifu 501-1193, Japan; orii.tadao@camel.plala.or.jp; 5 Department of Pediatrics, Thomas Jefferson University, Philadelphia, PA 19107, USA

**Keywords:** bone targeting drugs, osteoporosis, metabolic skeletal dysplasia, bisphosphonates, nanoparticles

## Abstract

The skeletal system, comprising bones, ligaments, cartilage and their connective tissues, is critical for the structure and support of the body. Diseases that affect the skeletal system can be difficult to treat, mainly because of the avascular cartilage region. Targeting drugs to the site of action can not only increase efficacy but also reduce toxicity. Bone-targeting drugs are designed with either of two general targeting moieties, aimed at the entire skeletal system or a specific cell type. Most bone-targeting drugs utilize an affinity to hydroxyapatite, a major component of the bone matrix that includes a high concentration of positively-charged Ca^2+^. The strategies for designing such targeting moieties can involve synthetic and/or biological components including negatively-charged amino acid peptides or bisphosphonates. Efficient delivery of bone-specific drugs provides significant impact in the treatment of skeletal related disorders including infectious diseases (osteoarthritis, osteomyelitis, etc.), osteoporosis, and metabolic skeletal dysplasia. Despite recent advances, however, both delivering the drug to its target without losing activity and avoiding adverse local effects remain a challenge. In this review, we investigate the current development of bone-targeting moieties, their efficacy and limitations, and discuss future directions for the development of these specific targeted treatments.

## 1. Overview

More than 350 disorders encompass the collective group of skeletal dysplasias. Diseases involving the skeletal system are particularly difficult to treat due to their complicated anatomical nature and the technical difficulties involving such a complex meshwork of different cell types, particularly in the avascular cartilage region. Despite the difficulties, targeting the skeletal system is critical for treatment of bone lesions. The skeletal system provides support, protects visceral organs, and enables movement. Defects in any component of this system can negatively impact theses critical functions. The idea of creating moieties that would allow for targeted delivery of pharmaceuticals to bone tissue first came in the late 1950s. Common skeletal disorders include osteoporosis, metabolic skeletal dysplasia, and infectious bone disease. It is difficult to treat most diseases of the skeletal system with non-targeted drug delivery. Visceral organs will utilize the bulk of the pharmaceutical drug, allowing very little to reach the bone. In addition, drugs are usually excreted before a significant amount can reach the bone. Higher doses are used to deliver small amounts of the drug into the bone; however, due to off-target drug absorption, this often results in adverse cytotoxic effects and significantly narrows the therapeutic options.

**Figure 1:**
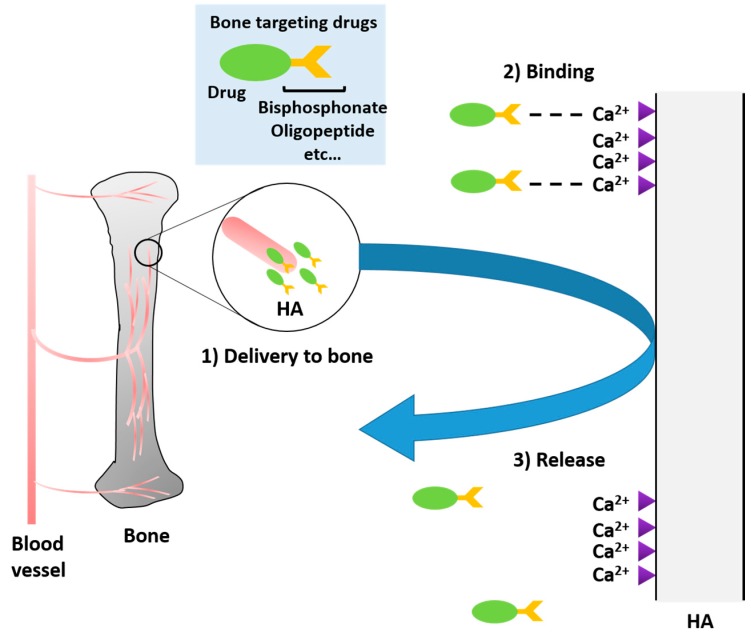
Scheme of delivery of bone-specific drug. HA, hydroxyapatite.

There are two general methods of targeted drug delivery to the skeletal system. The first involves targeting of the entire skeletal system (Figure 1). This can be accomplished through preferential binding to bone sites and can be done with either synthetic or biological moieties. Each of these has unique difficulties and benefits, but this general targeting is often sufficient to treat the pathogenesis of certain diseases including some metabolic skeletal dysplasias. The second general targeting strategy targets drugs to specific cellular locations within the skeletal system. These targets are often either osteoclasts, bone resorption cells, or osteoblasts, bone formation cells.

Many new systems for cell-specific bone targeting have been developed in the last few years, increasing drug stability, improving drug solubility, and preventing degradation to enable drugs to reach their targets before being eliminated in blood circulation. These targeting moieties can include synthetic components such as tetracycline and bisphosphonates (BPs) or biological components such as bone marrow stem cells [1,2]. Nanoparticles (NPs) are used as drug carriers, and new methods to couple drugs with the NPs can provide longer half-lives, greater efficacy, and specific binding properties [3,4].

In this review, we discuss different types of targeting moieties and the most recent advancements in targeting moiety coupling to provide efficient delivery. We also discuss how these targeting systems can be used in specific or common diseases of the skeletal system. 

## 2. Targeting Strategies

### 2.1. Bone-Targeting

#### 2.1.1. Bisphosphonates

Bisphosphonates (BPs) are a family of synthetic drugs that have been widely used for several decades. The first publication of BPs appeared in 1969, and they have had a significant impact on the clinical use of bone targeting drugs [2,5]. For a long time, BPs were considered as the gold standard in the delivery of drugs to the skeletal tissue. Their mechanism and structure are well understood [3,6] with two terminal phosphate bonds (P-C-P), and an easily accessible central carbon whose side chains can be manipulated. These side chains or R groups can influence drug release as well as binding interactions. BPs have an affinity for hydroxyapatite (HA) which is a main component in hard bone and causes preferential binding to this tissue [7]. As single agents, BPs inhibit the resorption of bone by osteoclasts and can simultaneously enhance osteoblast differentiation [8,9], thereby promoting bone formation. This has led to their widespread use in the treatment of osteoporosis. Limitations of adopting bisphosphonates as targeting agents include poor bioavailability that requires high doses, and side effects including ulcers, osteonecrosis of the jaw, and musculoskeletal pain [10,11]. 

##### Bisphosphonates to Target Proteins

Recent studies indicate that prolonged use of BP can have an inhibitory effect on osteoblasts, thereby, negatively impacting the quality of bone mass. Jaw osteonecrosis is estimated to affect 1 in 10,000 patients treated with BPs [12]. 

BPs are also used in the treatment of cancers that metastasize to the bone, and the very high doses of BPs needed to impair metastasis results in more frequent side effects [8,9,10,11]. Recently, carboxy-terminal collagen crosslinks (CTX) was discovered as an important biomarker that can accurately predict one’s risk of developing osteonecrosis of the jaw [12]. While this biomarker is useful in that it provides a threshold for risk analysis of BP treatment, its necessity is indicative that further efforts are required to reduce cytotoxicity to allow BPs to gain acceptance for use as targeting moieties to treat skeletal diseases other than osteoporosis.

A pharmacokinetic study with Alendronate (alendronic acid; ALN) modified proteins showed that the bone uptake of these modified proteins was, in fact, dependent on the specific modification. The results suggested that the known affinity of ALN alone is not sufficient to ensure bone targeting as smaller modified proteins were rapidly cleared from the circulation. When ALN modifications were used in conjunction with PEG modification (polyethylene glycol), plasma retention time for smaller proteins increased and more was targeted to bone [13].

##### Bisphosphonates to Target Nanoparticles

Great strides have been made in recent years in the targeted delivery of pharmaceuticals by way of nanoparticles (NPs). NPs are used to improve solubility and reduce the toxicity of drugs. Their small size allows flexibility in targeting and capability of reaching targets without inducing an immune response. Nano particles have been prevalent in treating bone metastasis, accelerating bone formation in osteogenesis and in conjunction with small RNAs as a vector for gene targeting and inhibition of bone loss. Typically, bisphosphonates are chemically coupled to polymeric glycolic acids that are then mixed with amphipathic polymers that create nanoparticles by non-covalent forces including Van der Waals and hydrogen bonding. Proteins, RNAs, and drugs can be incorporated into these nanoparticles. The efficiency of incorporation of compounds into NPs depends on their affinity for components of the NP. Drugs are often trapped in the hydrophobic core of NPs while hydrophilic agents such as proteins and RNA bind to the surface. 

In a recent study, the BP risedronate was bound to HA in methoxy poly(ethylene glycol)-poly lactic-co-glycolic acid (mPEG-PLGA) nanoparticles to treat an animal model of osteoporosis. This formulation reduced the toxicity of risedronate and improved its efficacy against osteoporosis by prolonging retention in the circulation [14]. This study clearly demonstrates that BPs can target nanoparticles to the bone. Salerno et al. developed a biodegradable compatible NP targeting system using poly (d,l) lactide—co-glycolic acid conjugated to alendronate containing the anti-cancer drug doxorubicin (DXR) [15]. Both prostate cancer and breast cancer metastasize to bone, causing considerable pain. Consequently, bone-targeted drugs may be more effective against metastatic cancer than conventional chemotherapy, with a fewer side effect. Breast cancer cell lines treated with DXR-loaded NPs showed a strong pattern of growth inhibition (*p* < 0.005). When the drug-loaded nanoparticles were administered systemically in a mouse model of bone metastasis, they were shown to target tumors at osteolytic sites. Both free DXR and DXR coupled to nanoparticles showed significant dose-dependent growth inhibition of tumor cell lines, although only the DXR loaded NPs were effective at dose 1 (580 ng/mL). Both DXR loaded NPs and unloaded NPs reduced the incidence of osteolysis, although the drug-loaded NPs were more effective [15]. It appears that the alendronate itself prevents osteolytic lesions in bone metastasis but does not directly affect tumor cell growth. 

#### 2.1.2. Tetracyclines 

Tetracyclines (TCs) are small molecular compounds that are traditionally utilized for their antibiotic properties to treat bacterial infections. TCs are also considered very useful in the development of targeting moieties aimed at treating diseases with skeletal manifestations particularly alveolar bone loss. Wang et al found that TC covalently bound to polycoglycolic acid (PLGA) biopolymer NPs enhances bone targeting of drugs due to a reaction between TC and hydroxyapatite (HA), a major constituent of bone tissue. This coupling combines the biocompatible byproducts of the PLGA biopolymer and the specific targeting of TC with NPs to enhance distribution and to limit cytotoxicity. Coupling of the TC to the biopolymer was confirmed using proton nuclear magnetic resonance (H-NMR) [16,17]. The NPs increased the amount of the osteogenic enhancing drug simvastatin (SIM) distributed to the skeletal system. Tetracycline coupled with PLGA NPs improved overall curative effects, which could reduce the drug dosage required for effective treatment. In addition, this system was found to reduce the drug distribution to off-target visceral organs as measured by fluorescent analysis [17,18].

TCs inhibit bone resorption by several mechanisms. In addition to targeting bone, TCs are also inhibitors of collagenases. They inhibit MMPs by chelating Ca^2+^ and Zn^2+^. Sequestration of Zn^2+^ prevents the conversion of procollagenase into its active form and indirectly down-regulates collagenase gene expression. TCs diminish acid production and the secretion of lysosomal cysteine proteinases. They also increase the number of active osteoblasts relative to inactive osteoblasts by increasing expression of procollagen mRNA. The biological function of tetracycline is not impaired by covalent binding to PLGA in NPs, allowing it to have continued effectiveness during bone differentiation [19].

#### 2.1.3. Polymeric Amino Acid Targeting

Oldberg and Heinegård found that several non-collagenous proteins that bind to HA had repeating sequences of acidic amino acid (Asp or Glu) [20]. Sekido et al. conjugated oligopeptides (Asp or Glu) to the fluorescent probe 9-fluorenylmethylchloroformate (Fmoc), and evaluated the affinity of these probes for HA both in vitro and in vivo [21]. The in vitro affinity for HA was dependent on the number of oligopeptide residue, and not on the optical isoform (l- or d-) or the acidic amino acid species (Asp or Glu). Pharmacokinetic analysis of the probes in mice showed that probes with six or more Asp residues were selectively delivered into the bone [21]. Thus, oligopeptide conjugation became a candidate carrier for bone targeting. To date, many preclinical studies have been conducted using oligopeptide conjugated drug for several diseases such as osteoporosis, infection disease, musculoskeletal disease, and cancer.

#### 2.1.4. Calcium Phosphate Biomaterials

Calcium phosphates (CaP) are a main component of bone, and are consequently potentially useful to deliver pharmaceuticals in bone. CaP scaffolds have been used to enhance healing after a bone fracture for decades, and the ease of synthesis and natural presence of CaPs in the human body have made such scaffolds a potential way to deliver drugs to the bone. CaP materials of several morphological distinctions including nanorods and nanoparticles can be synthetically prepared to include biological molecules before implantation into bone. Their high similarity to the bone mineral makes them both biocompatible and biodegradable [20]. However, synthesis of NaP nanostructured materials with well-defined morphology is a continuing challenge. In vivo testing of CaP vesicle-like nanospheres has suggested promising drug delivery with limited toxicity to cells [22]. This mechanism should play a beneficial role of CaPs in bone repair

### 2.2. Bone Cell-Specific Targeting 

#### 2.2.1. Osteoblast Targeting

Bone mass is regulated by a balance between bone resorption by osteoclasts derived during hematopoiesis and bone formation by osteoblasts derived from mesenchymal stem cells [23]. Osteoblasts are found in large numbers on the outside surface of bones. An imbalance in osteoblast and osteoclast differentiation can lead to skeletal disease [24]. There are several limitations of current drugs that enhance bone formation. Current clinical anabolic drugs include full-length parathyroid hormone (PTH 1–84) or its N-terminal fragment (PTH 1–34), but these drugs have severe side effects. There is, therefore, a great need for an osteoblast-targeted drug delivery system to treat osteogenic disorders with fewer side effects. 

Gene therapy is a promising strategy for effective and safe treatment of disease that delivers exogenous small nucleic acids such as DNA, RNA, small interfering RNA (siRNA) and micro RNA (miRNA). Clinical success for this approach has been limited due to nucleic acid biodegradation, lack of specificity and safe and effective delivery. The vector used to deliver nucleic acids can be either viral or non-viral. However, substantial challenges limit viral vector use, including safety and immunogenicity issues, promoting the development of non-viral vector delivery systems. While non-viral vectors can have lower immunogenicity, they tend to have more frequent off target effects and are often not biodegradable or biocompatible. 

Peptides are biocompatible, they have a low immunogenic effect, are easy to synthesize and can be produced at low cost. Peptides can be unstable during chemical modification and tend to denature when heated. Incorporation of the targeting moiety with NPs can improve stability and direct tissue distribution. Peptides have been used to target polyurethane (PU) nanomicelles that encapsulate small nucleic acid by electrostatic interactions. These conjugates have excellent biocompatibility and low toxicity making this an excellent osteoblast-targeted delivery system. An SDSSD-PU micelle NP has been used to deliver siRNA and miRNA to osteoblasts, exerting therapeutic activity by RNAi activity. SDSSD-PU was shown to target osteoblasts both in vivo and in vitro. Thus, SDSSD-PU NPs may be useful to treat osteoblast dysfunction that is typical seen in many skeletal diseases, including metabolic syndromes [25].

siRNAs play a role in the maintenance of homeostasis between osteoclasts and osteoblasts [26,27,28]. Concerns about the efficacy and safety of siRNA exist due to the dangers of off-target effects and the lack of osteoblast-specific delivery systems for such siRNAs. Liang et al. identified an aptamer that selectively recognized osteoblasts, and used this to target lipid nanoparticles containing an siRNA against Plekho1 to both rat and human osteoblasts [27]. There was significant osteoblast uptake of the siRNA resulting in osteoblast Plekho1 gene silencing. In vivo, the siRNA was targeted to the bone, and bone formation was significantly improved in osteopenic rats [27]. 

#### 2.2.2. Osteoclast Targeting 

The osteoclast absorbs bone tissue during growth and healing. The osteoclast is one of the most specialized human cells. Moreover, it is in careful homeostasis with the osteoblasts maintaining and repairing bone mass. Disturbances of the environment or regulation of osteoclast formation can affect skeletal homeostasis and lead to severe skeletal defects. Maintenance of a healthy human skeleton is in part dependent on mineralized bone matrix removal through bone resorption. This is accomplished by the osteoclast through identification of damaged bones and release of proteolytic enzymes and acidic oligopeptides. Without regulation of this function, the skeletal buildup of damaged bone matrix can cause skeletal malformation and physical clinical manifestation. Osteoclast targeting controls the maintenance of adequate bone mass throughout a person’s life span. Osteoclast targeting to reduce rates of bone resorption has been utilized in the treatment of bone metastasis, Paget’s disease, and osteoporosis [29,30]. 

#### 2.2.3. Targeting with Bone Marrow Stromal Cells (BMSCs)

Bone marrow stromal cells are a part of the adult skeleton cell population with strong osteogenic potential. The cells have high bioavailability and can be harvested readily from bone marrow of healthy unaffected individuals [31,32,33,34]. These cells have the potential to differentiate into chondrocytes, osteoblasts, adipocytes, etc. In the diseases that involve bone remodeling deficiencies and repair, these cells are, therefore, potentially useful in clinical value to deliver replacement enzymes to the bone of affected patients. 

BMSC-mediated gene-directed enzyme treatment has been used in anti-tumor therapy. In one study, a human gene was cloned from human hepatocytes and, through a constructed vector, was targeted into human BMSCs via liposomal transport. These transfected BMSCs were found to inhibit tumor growth by way of lymphoma cell apoptosis induction through the bystander effect when co-cultured with Raju cells [35,36]. In addition, inhibition of cross talk in BMSCs and certain cancer cells has been found to improve chemotherapy-induced toxicity. Pillinger et al. demonstrated this effect when acute myeloid leukemia cells were limited in their adhesion to BMSCs via VA4ACm fibronectin interactions, successfully limiting AML-BMSC adhesion. This shows that the drug resistance to AML chemotherapy, which is attributed to BMSCs, can be overcome with inhibition of adhesion in combination with standard chemotherapy agents [35]. Current efforts are focused on obtaining BMSCs from cord blood because although they are plentiful in cord blood, transplantation from such a source to an adult is still severely limited, although sibling matched donors have achieved greater success [31,37,38].

## 3. Limitations and Perspective

The concept of bone targeting started at the beginning of last century and has tremendoushuge potential in the treatment of skeletal disorders. Non- targeted treatments cause problems because they deliver the majority of the drugs into visceral organs. Most problems are caused by lack of bioavailability and biocompatibility as well as poor circulation time [39,40,41,42]. While targeting moieties are improving drastically with the advent of NP-based delivery components and cell-specific targeting, off-target negative side effects remain in each delivery system. In addition, the risk must be measured individually for each patient, as these moieties can be detrimental in immunocompromised or bone density deficient patients. Specific examples of limitations for targeting systems are shown in Table 1.

## 4. Impact and Significance of Bone Targeting on Specific Disease Burdens 

### 4.1. Infectious Disease

#### 4.1.1. Acidic Oligopeptide-Modified Drug for Infectious Disease

Osteomyelitis is infection and inflammation in the bone caused by acute events such as bone fracture, surgical intervention, and bone injury. This disease can also be caused by hematogenous spread after bacteremia. *Staphylococcus aureus* is the major phlogogenic bacteria in osteomyelitis. In general, antibiotic therapy is used as a treatment for osteomyelitis for a few weeks to several months. In severe cases, however, surgical debridement is required. Although the concentration of antibiotics needed for treatment of bone lesions must be maintained at a very high level for a long period, this sustained exposure increases the risk of adverse effects.

Takahashi et al. found that conjugation of quinolones to an acidic oligopeptide worked as a bone targeting carrier in a mouse model. The selective bone delivery of quinolones conjugated to an acidic oligopeptide was effective in treating osteomyelitis, reducing inflammatory destruction and eventual necrosis of the healthy bone [53]. Levofloxacin (LVFX) is a fluoroquinolone antibiotic with a wide spectrum of activity against both Gram-positive and Gram-negative bacteria. It functions by inhibiting Type II topoisomerase enzymes. Takahashi et al. conjugated a six aspartic acid oligopeptide to LVFX (LVFX-D_6_) to selectively deliver this quinolone into bone lesions and investigated pharmacokinetic and pharmacological studies of LVFX-D_6_ in a mouse model of osteomyelitis [53]. After intravenous injection of LVFX-D_6_ in a normal mouse, the concentration of LVFX-D_6_ stayed higher in bone and bone marrow for one week when compared to that of un-modified LVFX. In all tissues except the kidney, the bone to plasma ratio (K_p,app_) of LVFX-D_6_ was increased when compared with that of LVFX at 2 h after injection, indicating that LVFX-D_6_ was selectively delivered into bone and bone marrow. To evaluate the pharmacological effect of LVFX-D_6_ and LVFX, these quinolones were administered into a mouse model of osteomyelitis inoculated with *Staphylococcus aureus* into tibia bone. LVFX-D_6_ continued to suppress the proliferation of the bacteria for at least for 6 days while a colony-forming unit in LVFX treated mice was almost recovered to the levels of untreated mice by 6 days after injection*.*

#### 4.1.2. BP-Modified Drug for Infectious Disease

Since BPs have a high affinity for the calcium ion of HA, BP-modified antibiotic agents for osteomyelitis have been examined in a pre-clinical study [47,48,49]. PLGA-PEG NPs have been used for drug delivery due to the merits of the PLGA-PEG moiety previously discussed. A 4,5-Dimethylthiazol-2-yl)-2,5-diphenyltettrazolium bromidefor assay or MTT assay showed that antibiotic-loaded PLGA-PEG-ALN micelles were not more cytotoxic than blank micelles in rBMSCs L02 cells, indicating that vancomycin-loaded PLGA-PEG-ALN micelles would be safe in the treatment of osteomyelitis. Antibacterial effects of this bone targeting micelle were also tested in vitro by using *Staphylococcus aureus*. Although the minimum inhibitory concentration (MIC) of free vancomycin was 2 µg/mL, the MIC of vancomycin-loaded PLGA-PEG-ALN micelles was higher, at 16 µg/mL. Authors explained that free drug concentration in the medium did not reach the MIC when the concentration in the conjugate was lower than 16 µg/mL. However, this micelle formulation did inhibit the activity of *Staphylococcus aureus* in the in vivo model of osteomyelitis [46,47].

Ferreira et al. developed long-circulating and ALN-conjugated liposomes containing ^99m^technetium (^99m^Tc)-radiolabeled ceftizoxime to improve the efficiency of drug delivery into the infectious site of bone [43]. Ceftizoxime is a third-generation cephalosporin which has wide broad spectrum activity. Previously, authors have developed pH-sensitive liposome containing ^99m^Tc-radiolabeled ceftizoxime to diagnose infection disease in bone lesions. However, uptake of this liposome containing ^99m^Tc-radiolabeled ceftizoxime in the infectious area was not higher than free ^99m^Tc-radiolabeled ceftizoxime. Scintigraphic image test showed that ALN-decorated liposome contacting ^99m^Tc-radiolabeled ceftizoxime increased target-to-non-target ratio in tibia compared with the non-decorated formulation or the non-long circulating formulation in an osteomyelitis animal model. Target-to-non-target ratio in the tibias of the osteomyelitis model was also higher than that in an aseptic inflammation model or healthy animal model. Thus, the authors suggested that this bone-targeting liposome containing ^99m^Tc-radiolabeled ceftizoxime showed high affinity in infectious foci of bone lesions and would be useful for diagnosis and treatment of osteomyelitis [43].

### 4.2. Osteoporosis 

#### 4.2.1. Acidic Oligopeptide-Modified Drug for Osteoporosis

Osteoporosis is a metabolic disorder of bone strength characterized by low bone mass and micro-architectural deterioration of bone tissue. This disease leads to increasing bone fragility and fracture risk. Pathophysiology of osteoporosis is based on the weakening of bone bridge formation due to relative increasing bone resorption against ossification. Osteoporosis is a serious problem particularly for women, and a major cause of decreasing bone mineral density in this disorder is acute hypo secretion of estrogen with the menopause. Hormone-related therapy for osteoporosis, i.e., estrogen, maintains bone mineral density. However, this therapy can increase the risk of breast cancer, endometrial cancer, blood clots and heart disease. Therefore, development of bone-targeting therapy for osteoporosis is required not only to enhance the treatment effect but also to reduce the risk of such adverse effects.

Sekido et al. and Yokogawa et al. performed a pharmacokinetic analysis of estradiol (E2) conjugated with l-Asp-hexapeptide at either the 17β-position (E2-17 βD_6_) or the 3-position (E2-3D_6_) via succinate ester [44,45]. After a single intravenous administration of 3.7 µmol/kg E2, E2-17 βD_6_ or E2-3D_6_ in normal mice, total clearance (CL_tot_) of both oligopeptide-conjugated forms of E2 was significantly decreased compared with that of free E2, and apparent tissue to plasma concentration ratio (K_p,app_) of E2-17 βD_6_ or E2-3D_6_ in femur was markedly higher than that of E2 while K_p,app_ of both oligopeptide-conjugated E2 in other tissues was unchanged or slightly decreased. These results indicate that oligopeptide-conjugated E2 could be selectively delivered to bone lesions. The affinity of E2-17 βD_6_ or E2-3D_6_ to human estrogen receptor (ERα and ERβ) was 10,000-fold or 100-fold less than that of E2, respectively [44,45]. Pharmacological analysis showed administration of E2-17 βD_6_ or E2-3D_6_ in ovariectomized murine model improved bone mineral density without increasing uterine weight although both bone mineral density and uterine weight were elevated after injection of E2. Therefore, acidic oligopeptide-conjugated E2 would be effectively delivered into the bone lesion and reduce the risk of side effect of E2.

#### 4.2.2. BP-Modified Drug for Osteoporosis

BP-conjugated prodrugs of prostaglandin E_2_ [54] and 17β-estradiol [43,46,55] were developed in preclinical studies. These studies showed that different types of linkages affected pharmacokinetics and pharmacological effects of the BP-conjugated drug. As new targeting techniques for bone, preparation of BP conjugated to polymers, NPs or other materials are under investigation for an anti-osteoporotic drug [56,57]. Katsumi et al. reported that PEG-conjugated-ALN improves bioavailability and safety since oral bioavailability of free ALN is low and it causes mucosal damage as an adverse effect [57]. Intrapulmonary administration of PEG-conjugated-ALN in rats did not induce pulmonary mucosal damage or affect drug absorption when compared to free ALN. Intrapulmonary administration of PEG-conjugated-ALN in an osteoporotic rat model slowed decrease of growth plate to a similar level achieved with ALN. The effects were evaluated on bone tissue from the right tibia of female rats with treatment groups including intrapulmonary administration of alendronate at a dosage of 2 mg/kg and intrapulmonary administration of PEG alendronate at a dosage of 2 mg/kg. Groups were evaluated after eight weeks, and both the width of the growth plate and the density of the bone structure were reduced, with the intrapulmonary administration of PEG alendronate preventing or reversing the decreases in the width of the bone plate and the density of the bone structure. 

For the future, linkage of BP to imaging agents within NPs and polymers would enable bone monitoring and diagnosis for osteoporosis [58].

### 4.3. Rheumatoid Arthritis 

Rheumatoid arthritis (RA) is a chronic systemic inflammatory disease. Inflammation from the synovial cell and endothelial cell activation leads to cartilage and bone destruction by causing the excessive production of cytokines, chemokines and other factors including TNF-a, IL-6, and IL-1. Recently, several reports showed high-mobility group box 1 (HMGB1) level was elevated in serum and synovial fluid of RA patients [50,59]. HMGB1 binds to a receptor for advanced glycation end products (RAGE) [60,61] that activates intracellular signaling and produces inflammatory cytokines [43]. Takahashi et al. conjugated an acidic oligopeptide to endogenous secretory RAGE (esRAGE) as a decoy receptor. They determined the tissue distribution of this bone-targeting endogenous secretory receptor and its effect on RA in an animal model [56]. After administration of acidic oligopeptide conjugated-esRAGE (D_6_-esRAGE, 1 mg/kg) to an RA model mouse, they showed that D_6_-esRAGE was retained in bone lesions for at least a week while esRAGE was not detectable after 72 h. Weekly administration of D_6_-esRAGE suppressed serum level of inflammatory cytokines such as TNF-a, IL-1, and IL-6, and cartilage and bone destruction was less pronounced than in esRAGE treated RA mice. D_6_-esRAGE showed a histopathological score of less than 1.5 in the synovial lesion, less than 1.0 in cartilage destruction and 0.9 in bone destruction. The untreated controls showed histopathological scores of 2.75 in synovial lesions, 2.0 in cartilage destruction and 2.25 in bone destruction. D_6_-esRAGE and esRAGE significantly decreased plasma levels of TNF-a, IL-1, and IL-6, as measured by an enzyme-linked immunosorbent assy (ELISA) (*p* < 0.05) however D_6_-esRAGE was more effective than free esRAGE [53].

### 4.4. Metabolic Skeletal Dysplasia 

Metabolic disorders are caused by an inborn error of metabolism due to an inherited gene mutation. Some metabolic disorders occur from birth; others only become apparent in later life. The result of the mutated or deficient gene is typically a build of- material that should be degraded and a lack of material that is necessary for the metabolic pathway to function correctly, usually caused by enzyme deficiency [51,62,63,64,65,66,67]. Often these metabolic disorders will have a skeletal component. One of the most common genetic metabolic disorders that lead to skeletal dysplasia is Mucopolysaccharidosis (MPS). Although this group of disorders can be caused by the mutation of different genes and have different modes of heritability, all forms of MPS result in a deficiency of specific enzymes necessary to degrade GAG proteins. The lack of the enzyme leads to unique clinical phenotypes, in which one of the most recognizable features is stunned long bone growth and consequent abnormal skeletal dysplasia. 

Enhancement of the effects of the deficient enzyme treatment involves targeting the enzyme directly to the bone. Attempts at treating skeletal dysplasia in MPS involving non-bone targeting delivery has failed to produce skeletal improvements due to the short circulation time and its excretion and uptake in non-target visceral organs. Enzyme replacement therapy (ERT) for Mucopolysaccharidosis type IVA (MPS IVA) provides a greater effect on bone pathology in MPS IVA mice when coupled with the acidic oligopeptide tag although early treatment is recommended for the greater success [64].

Nishioka et al. found that tissue-nonspecific alkaline phosphate (TNSALP) tagged with an acidic oligopeptide could improve hypophosphatasia when this enzyme was deficient in vivo. This method resulted in a 30-fold higher affinity for HA relative to the affinity of the untagged enzyme [51]. This bone targeting ALP enzyme showed a substantial clinical improvement in bone mineral density, prolonged life span, and activity of daily living of patients with hypophosphatasia and the modified enzyme was approved by the Federal Drugs Agency (FDA) to treat hypophosphatasia [52].

Gene therapy, which would provide a permeant and one-time solution, could be effective if administered before the onset of skeletal dysplasia; however, a vector system that would allow the delivery is not yet available in a clinical setting. Gene therapy is expected to be a one-time permanent treatment, with the protein of interest continuously secreted from transduced cells. We have already engineered an adenoassociated virus (AAV2) vector to target gene delivery to the bone by integrating multiple copies of a short acidic amino acid peptide into the gene vector capsid. To increase the vector affinity for HA, eight aspartic acidic residues (D8) were inserted immediately after the N-terminal region of the VP2 capsid protein in the packing plasmid. The modified vector was generated with physical titers and transduction efficiencies comparable to the unmodified vector. After intravenous administration of this modified vector capsid into the MPS IVA model mouse, the bone-targeting vector showed significantly higher HA affinity and vector genome copies in bone compared with the unmodified vector. Expression of N-acetyl-galactosamine-6-sulfate (GALNS) in bone was also significantly elevated compared with enzyme levels in bone of mice transduced with the unmodified vector [68,69].

## 5. Future Directions

Bone-targeting systems to deliver siRNA have been designed, and their effects in the treatment of osteoporosis have been examined in a preclinical study [48,70,71]. It is difficult to deliver siRNA into targeted cells since the water soluble siRNA cannot cross the hydrophobic plasma membrane. D8 bone-affinity polymeric NPs packaged with siRNA for *semaphorin 4d* was administered into osteoporotic mouse model via weekly intravenous injection. After administration, the number of active osteoblasts at the bone surface was significantly increased, and bone mass density (BMD) and bone volume/tissue volume were improved compared with those in untreated mice [70].

Manipulation of dysregulated miRNA in osteoclasts has been found to facilitate microRNA modulators with great success. Dang et al. found that when miRNA modulators are coupled with d-(Asp)s, antogomiR-148a is enriched, resulting in the downregulation of osteoclast forming miR0148a in osteoclasts [49]. This leads to reduced bone resorption and attenuated deterioration of trabecular structure in model osteoporotic mice. While this specific approach has not been attempted in human subjects, its success in vivo with limited off-target effects shows promise.

In the future, combining bone-targeting with gene therapy could significantly improve efficacy in the treatment of bone diseases. However, type of vector, route of administration and adverse effects are yet to be determined.

## 6. Conclusions

Although the idea of bone-targeted delivery of pharmaceuticals in skeletal disorders has been studied for decades, the identification of carrier systems capable of increasing efficacy, reducing the off-target effect, minimizing delivery to visceral organs, and allowing increased solubility, leading to maintenance of bone integrity, have only recently been examined in detail. The evaluation of these therapies needs to include risk as well as cost/benefit assessments. These factors along with problems of bioavailability remain as challenges for this type of delivery system. Nevertheless, the advancement of some targeting moieties into clinically approved drugs and advances in gene therapy offer the prospects of developing successful one-time treatments to correct bone defects in metabolic skeletal dysplasia. 

## Figures and Tables

**Table 1:** Examples of moieties for specific targeting in the treatment of skeletal disorders.

Targeting Moieties	Targeted Tissues	Specific Drugs and Moieties	Primary Limitations
Bisphosphonates [12,43,44,45]	All skeletal tissues	samarium-153, pamidronate, alendronate, risedronate	Cytotoxicity, osteonecrosis
Tetracycline derivatives [16,17,18]	All skeletal tissues	Estradiol	Lack of specificity, gastrointestinal distress
PLGA USAuNPs [19,46,47]	Cell-specific	Under review	Lack of understanding in mechanism, size restriction, inflammatory response
CKIP-1 [48,49]	Osteoblasts	CkIP-1 siRNA	Off target drug delivery and circulation time limitations
l-(asp)_6_, l-(glu)_6_ [50,51,52]	All bone tissue	CD6-TNSALP, CD8-TNSALP	Nonnative functioning, inadequate bone delivery
Calcium Phosphates (CaP’s) [26,31]	Resorption surfaces	b-TCP + copolymers PLA-DX-PEG,ACP/PLLA nanofibrous scaffold	Calcium toxicity from overabundance, osteosarcoma
SDSSD [24,25,26,27]	Osteoblasts	PTH 1-84, PTH 1-34	Circulation time limitations
BMSC specific aptamer [35,36]	BMSCs	miR-188-3p antagomir	Circulation time limitations
